# The association between objectively measured physical activity and home blood pressure: a population-based real-world data analysis

**DOI:** 10.1038/s41371-025-01014-8

**Published:** 2025-04-03

**Authors:** Minako Kinuta, Takashi Hisamatsu, Kaori Taniguchi, Mari Fukuda, Noriko Nakahata, Hideyuki Kanda

**Affiliations:** 1 Department of Public Health, Okayama University Graduate School of Medicine, Dentistry and Pharmaceutical Sciences, Okayama, Japan; 2 Department of Environmental Medicine and Public Health, Izumo, Shimane University Faculty of Medicine, Izumo, Japan; 3 Department of Health and Nutrition, The University of Shimane Faculty of Nursing and Nutrition, Izumo, Japan

**Keywords:** Lifestyle modification, Risk factors

## Abstract

Few studies have examined the association of objectively measured habitual physical activity (PA) and sedentary behavior with out-of-office blood pressure (BP). We investigated the associations of objectively measured PA intensity time, sedentary time, and step count with at-home BP. Using accelerometer-recorded PA indices and self-measured BP in 368 participants (mean age, 53.8 years; 58.7% women), we analyzed 115,575 records of each parameter between May 2019 and April 2024. PA intensities were categorized as light (2.0–2.9 metabolic equivalents [METs]); moderate (3.0–5.9 METs); vigorous (≥6.0 METs), or sedentary (<2.0 METs): the median [interquartile ranges] for these variables was 188 [146–232], 83 [59–114], 1 [0–2], 501 [428–579] minutes, respectively, and for step count, was 6040 [4164–8457]. Means [standard deviations] for systolic and diastolic BP were 116.4 [14.2] and 75.2 [9.3] mmHg, respectively. A mixed-effect model adjusted for possible confounders showed that 1-h longer in vigorous PA was associated with lower systolic and diastolic BP (−1.69 and −1.09 mmHg, respectively). A 1000-step increase in step count was associated with lower systolic and diastolic BP (−0.05 and −0.02 mmHg, respectively). Associations were more pronounced among men and participants aged <60 years. Sedentary time was positively associated with BP in men and participants aged <60 years, but inversely associated with BP in women and participants aged ≥60 years. Our findings suggest that more PA and less sedentary behavior were associated with BP reduction, particularly among men and participants aged <60 years. However, the clinical relevance of this effect remains uncertain because of its modest magnitude.

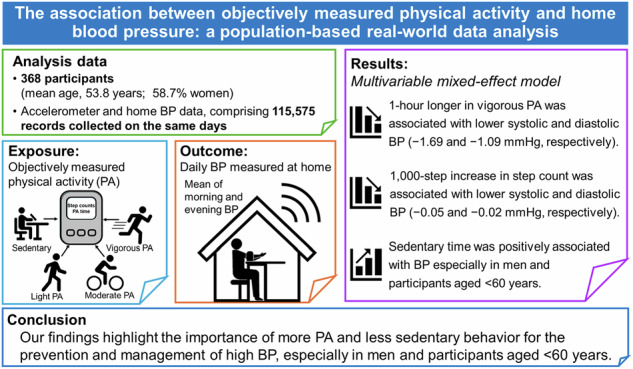

## Introduction

Hypertension is one of the most important modifiable risk factors for cardiovascular disease (CVD). The prevalence of elevated blood pressure (BP) remains high, with the greatest absolute burden of elevated BP particularly in the East Asian and Pacific regions [1, 2]. Recent international hypertension management guidelines confer increasing importance to methods of measuring BP outside the medical office (e.g., self-measured BP at home) for assessment of CVD risk [3–5]. The major advantage of out-of-office BP (e.g., home BP) measurement is that it provides a large number of BP measurements while minimizing the white-coat effect and observer bias, facilitating a highly reliable assessment of actual BP [6–8]. Indeed, home BP measurement is superior to conventional office BP in predicting CVD [6, 9].

Physical activity (PA) is commonly recommended as an important lifestyle modification that may aid in the prevention of hypertension [10]. Several previous studies report that moderate-to-vigorous PA is associated with lower office BP and reduced hypertension risk [11, 12]. The Prospect Physical Activity, Sitting and Sleep consortium (ProPASS) conducted a cross-sectional study examining associations between 24-h behavior compositions and BP. This study demonstrated that adding just 5 min of exercise-like activity (e.g., running and cycling) was associated with reductions in systolic BP (SBP) (0.68 mmHg) and diastolic BP (DBP) (0.54 mmHg) [13]. Additionally, higher levels of sedentary behavior are associated with higher BP levels [14, 15] and higher risks of CVD [16] and mortality [17]. The 2018 Physical Activity Guidelines Advisory Committee graded the evidence that sedentary behavior was associated with mortality and CVD as ‘strong’ [18] and added a recommendation to ‘sit less and move more’ to the 2018 federal Physical Activity Guidelines [19]. However, the majority of previous studies have assessed PA and sedentary behavior based on self-reported questionnaires in relation to office BP. By contrast, few studies have examined objectively measured habitual PA or daily step count and out-of-office BP [20]. Furthermore, little is known regarding the effect of objectively measured PA intensity (i.e., light, moderate, and vigorous PA and sedentary behavior) on out-of-office BP.

The Masuda Study is an ongoing prospective cohort study being performed to monitor daily trends in home BP measured using Internet of Things (IoT) technologies and PA measured using a tri-axial accelerometer on the same day among community-dwelling individuals aged 20 to 74 years [21, 22]. Using longitudinal, real-world data obtained during 5 years of the Masuda Study (May 2019 to April 2024), we examined the associations of objectively measured time spent in different PA intensities (i.e., light, moderate, and vigorous), sedentary time, and step count with self-measured BP at home. In Japan, home BP measurement is widespread, while the prevalence of hypertension is higher and the control rate of hypertension is lower than in Western countries [1, 3, 23]. Therefore, understanding the relationship between objectively measured daily PA and BP might yield fruitful targets to evaluate for the prevention and control of high BP.

## Methods

### Study participants

The Masuda Study is an ongoing prospective cohort study being performed to monitor daily trends in lifestyle-related factors including BP, dietary nutrition, and physical activity using IoT technologies among community-dwelling individuals aged 20 to 74 years in Masuda, Shimane, Japan [21, 22]. From May 2019 to April 2024, 483 men and women agreed to participate in the survey and provided written informed consent; they then used a tri-axial accelerometer to measure PA and step counts, performing home BP measurements on the same day. Inclusion criteria for this study were as follows: participants who (1) had no missing covariates (*n* = 41 excluded); (2) were free of a history of CVD (*n* = 46 excluded); (3) took between 500 and 20,000 steps per day [24] (*n* = 2 excluded); (4) wore the accelerometer for at least 10 h per day [25] (*n* = 9 excluded) and; (5) who measured at least 5 valid days during the study period (*n* = 17 excluded). Thus, a total of 368 participants (mean age, 53.8 years; 58.7% women) with accelerometer and home BP data comprising 115,575 records each of PA, step counts, and home BP measurements on the same days were analyzed for the present study. The institutional review board committee of Okayama University Graduate School of Medicine, Dentistry and Pharmaceutical Sciences and Okayama University Hospital approved the study (approval number: 2002-042). The study followed the code of ethics of the World Medical Association (1975 Declaration of Helsinki).

### Measurements of physical activity

Objectively measured time spent in different PA intensities (i.e., light, moderate, and vigorous), sedentary time, and step counts were obtained using a validated tri-axial accelerometer (OMRON Active style Pro HJA-750C; OMRON Healthcare Co., Ltd., Kyoto, Japan) [26]. Excluding the time during swimming and bathing, the subjects were asked to wear the accelerometer on their waist. The device, a Micro Electro Mechanical Systems-based triaxial accelerometer, measures 52 × 40 × 12 mm and weighs approximately 23 g, including the battery. Triaxial acceleration is recorded with a sensitivity of 3 mG at a sampling rate of 32 Hz. Each of the three signals from the triaxial accelerometer is processed through a high-pass filter with a cut-off frequency of 0.7 Hz to remove gravitational acceleration components. The integral of the absolute value of each of the three axes’ acceleration signals is calculated over 10-second intervals. The device then applies three specific equations to calculate PA intensity based on the type of activity, as previously validated in previous studies [26–28]. Metabolic equivalent (MET)-based criteria were used to determine the intensity of activities: <2.0 METs for sedentary behavior [29], 2.0–2.9 METs for light PA, 3.0–5.9 METs for moderate PA, and ≥6.0 METs for vigorous PA [30]. PA and sedentary behavior variables were summed across each adherent day (defined as ≥10 h of wear) [25].

### Measurements of home BP

Self-measured home BP values were obtained using a validated automated device (OMRON HEM-9700T; OMRON Healthcare Co., Ltd., Kyoto, Japan) [31]. All participants were instructed to place a cuff of appropriate size on the same arm throughout the measurements and to measure their BP in a sitting position after ≥2 min of rest according to the Japanese Society of Hypertension 2019 guidelines for the Management of Hypertension (JSH 2019) [3]. Two home BP readings were taken at 30-second intervals in a sitting position in the morning and evening every day. Morning BP was measured within 1 h of waking, after urination, before breakfast, and before taking antihypertensive medication. Evening BP was measured before going to bed, and the participants were instructed to avoid measuring their BP immediately after taking a bath, drinking alcohol, or smoking. The morning and evening BP data were automatically transmitted via the Internet and stored in the cloud server. We defined morning BP values as those measured between 3:00 a.m. and 10:59 a.m. and evening BP values as those measured between 5:00 p.m. and 1:59 a.m., in accordance with previous studies [22]. We included participants who conducted home BP measurements for at least 5 days during the study period [3], using BP values obtained on days when the accelerometer was worn. Morning and evening home BP were calculated as the mean of the two BP measurements, respectively. Daily home BP was the average of the mean BP in the morning and evening, respectively.

### Covariate assessment and statistical analysis

A self-administered questionnaire was used to obtain information on demographics, smoking habits, alcohol drinking, medication use, and medical history. After participants had completed the questionnaires, trained research staff confirmed all responses with the participants. Body mass index (BMI) was calculated as weight (kg) divided by height squared (m^2^).

Continuous variables are expressed as mea*n* ± standard deviation or median (interquartile range), and categorical variables are presented as the number (percentage) of individuals. Continuous normally distributed variables were compared using the Student’s t-test; differences between non-normally distributed variables were tested with the Mann–Whitney U test. Categorical variables were compared using the chi-square test. The associations between home BP and time spent in different PA intensities, sedentary time, and step count were investigated by applying a mixed-effects model based on our longitudinal data. Adjustments were made for age, sex, BMI, smoking status, alcohol drinking, diabetes mellitus, and antihypertensive medication use. These covariates were selected a priori because they have known correlations with PA, sedentary behavior, and high BP [32]. Additionally, sedentary time was adjusted for in the analysis of moderate and vigorous PA and step count, while time spent in moderate and vigorous PA was adjusted for in the analysis of light PA and sedentary time [25]. To account for the potential influence of individuals with 0 min of vigorous PA on the regression slope, we categorized vigorous PA time into the following groups: 0 min (reference), 1 min (50th percentile), 2–4 min (75th percentile), and 5 or more minutes (90th percentile) and then examined the association of categories of vigorous PA time with BP by entering the median values of each category using the adjusted mixed-effects model. For sensitivity analyses, we conducted stratified analyses by age (<60 and ≥60 years), sex, antihypertensive medication use, obesity status (BMI < 25 and ≥25 kg/m^2^), and hypertension status (home BP ≥ 135/85 mm Hg or antihypertensive medication use) [3]; we further tested for multiplicative interactions between these factors and PA indices (time spent in different PA intensities, sedentary time, and step count) in relation to BP levels. Analyses were performed using a statistical program (STATA version 17.0; StataCorp LP, College Station, TX, USA). Two-tailed *P-*values of <0.05 were considered statistically significant.

## Results

Table 1 shows the characteristics of the participants. The means [standard deviations] of SBP) and DBP were 116.4 [14.2] mmHg and 75.2 [9.3] mmHg, respectively, during the study period. The median [interquartile range] daily equipment time of the tri-axial accelerometer was 780 [688–877] minutes. The median times for light, moderate, and vigorous PA and sedentary time were 188 [146–232], 83 [59–114], 1 [0–2], and 501 [428–579] minutes, respectively. The median step count was 6040 [4164–8457]. Of the 368 participants, 96 (26.1%) took antihypertensive medication. The mean BMI was 23.0 [3.6] kg/m^2^. The participants’ characteristics stratified by sex and age (<60 and ≥60 years**)** are shown in Supplementary Tables 1 and 2, respectively.

**Table 1:** Baseline characteristics of participants in the Masuda Study (2019–2024).

Variables	Total (n = 368)
Age, years	53.8 ± 11.8
Women	216 (58.7)
Body mass index	23.0 ± 3.6
SBP, mmHg	116.4 ± 14.2
DBP, mmHg	75.2 ± 9.3
Equipment time of tri-axial accelerometer, min/day	780 (688–877)
Light	188 (146–232)
Moderate	83 (59–114)
Vigorous	1 (0–2)
Sedentary time, min/day	501 (428–579)
Step count, day	6040 (4164–8457)
Current smoker	29 (7.9)
Alcohol drinking	236 (64.1)
Antihypertensive medication use	96 (26.1)
Diabetes mellitus	20 (5.4)

Data are presented as mean ± standard deviation, n (%), or median (interquartile range).

*SBP* systolic blood pressure, *DBP* diastolic blood pressure.

**Fig. 1:**
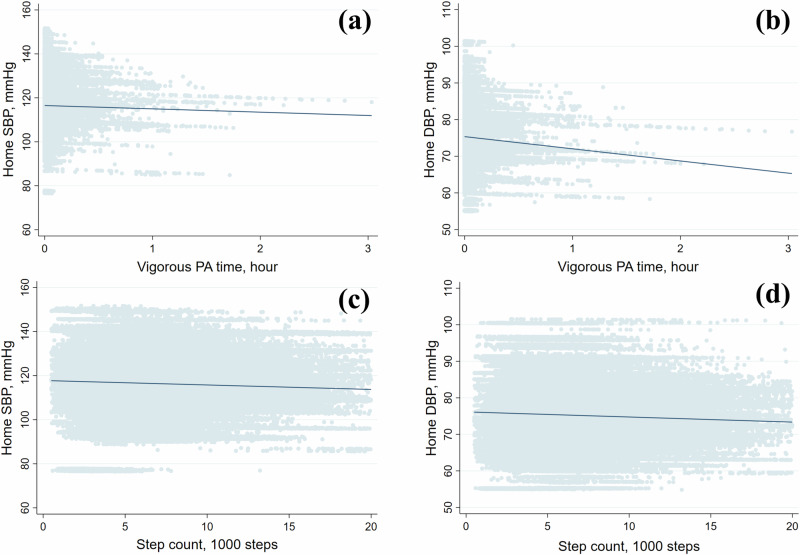
Associations of vigorous PA time and step count with home blood pressure. Associations of vigorous PA time with home SBP **(a)** and DBP **(b)**, and step count with home SBP **(c)** and DBP **(d)**. Based on data from 368 participants with accelerometer-recorded PA, step count, and home BP measurements on the same day, a total of 115,575 records were analyzed. The mixed-effect model was adjusted for age, sex, body mass index, smoking status, alcohol drinking, diabetes mellitus, antihypertensive medication use, and sedentary time. *SBP* systolic blood pressure, *DBP* diastolic blood pressure, *PA* physical activity.

The associations of PA, sedentary time, and step count with home BP analyzed using the multivariable-adjusted mixed-effects model are shown in Table 2 and Fig. 1. A 1-h longer vigorous PA time was associated with lower SBP and DBP by 1.69 and 1.09 mmHg, respectively. Similarly, every 1000-step increase in step count was associated with lower SBP and DBP by 0.05 and 0.02 mmHg, respectively. Light PA time was positively associated, while sedentary time was inversely associated, with SBP. The associations of moderate PA time with SBP and DBP did not demonstrate a clear trend. In additional analysis categorizing vigorous PA time was, compared to the reference category (0 min), SBP was lower by 0.06 mmHg, 0.19 mmHg, and 0.30 mmHg for 1 min, 2–4 min, and 5 or more minutes of vigorous PA, respectively (P for trends, 0.009). Similarly, DBP was lower by 0.11 mmHg, 0.09 mmHg, and 0.20 mmHg, respectively (P for trends, 0.015) (Supplementary Table 3).

**Table 2:** Association of physical activity, sedentary time, and step count with home blood pressure.

	SBP (mmHg)		DBP (mmHg)	
	*Β* (95% CI)	*P*-value	*Β* (95% CI)	*P*-value
Light PA time, hour	0.19 (0.11 to 0.27)	<0.001	0.04 (−0.01 to 0.09)	0.109
Moderate PA time, hour	0.02 (−0.08 to 0.12)	0.684	−0.05 (−0.11 to 0.02)	0.170
Vigorous PA time, hour	−1.69 (−2.24 to −1.13)	<0.001	−1.09 (−1.45 to −0.74)	<0.001
Sedentary time, hour	−0.04 (−0.07 to −0.002)	0.039	0.01 (−0.01 to 0.03)	0.415
Step count, 1000 steps	−0.05 (−0.07 to −0.03)	<0.001	−0.02 (−0.04 to −0.01)	<0.001

Analysis of moderate and vigorous PA time and step count was adjusted by age, sex, body mass index, smoking status, alcohol drinking, diabetes mellitus, antihypertensive medication use, and sedentary time; analysis of light PA and sedentary time was adjusted by age, sex, body mass index, smoking status, alcohol drinking, diabetes mellitus, antihypertensive medication use, and time spent in moderate and vigorous PA.

*CI* confidence interval*, SBP* systolic blood pressure, *DBP* diastolic blood pressure, *PA* physical activity.

The results of the sex-stratified analysis (Supplementary Table 4) show that inverse associations of vigorous PA time or step count with SBP and DBP were more pronounced among men than among women. Longer sedentary time was associated with higher SBP and DBP in men. On the other hand, in women, light PA time was positively associated, while sedentary time was inversely associated with SBP and DBP.

The results of the age-stratified analysis (Supplementary Table 5) indicate that inverse associations of moderate PA time, vigorous PA time, and step count with SBP and DBP were more evident in participants aged <60 years than in those aged ≥60 years. Longer sedentary time was associated with higher SBP and DBP especially in those aged <60 years. Whereas, sedentary time was inversely associated with SBP and DBP in those aged ≥60 years.

In the analysis stratified by antihypertensive medication use (Supplementary Table 6), light PA time was positively associated, while sedentary time was inversely associated, with SBP and DBP especially among participants not taking antihypertensive medication. Sedentary time was positively associated with SBP and DBP among those taking antihypertensive medication. Negative associations of vigorous PA time and step count with SBP and DBP were not dependent on antihypertensive medication use. In the analysis stratified by obesity status (Supplementary Table 7), there were tendencies for more pronounced positive associations of light PA time, and more pronounced inverse associations of moderate or vigorous PA time, with SBP or DBP among obese participants compared with non-obese participants. In the analysis stratified by hypertension (Supplementary Table 8), overall, there was a tendency for more pronounced inverse associations of moderate or vigorous PA time with BP especially among participants with hypertension. Meanwhile, light and moderate PA time tended to show positive associations with BP among participants without hypertension.

## Discussion

We analyzed the 5-year population-based real-world data of time spent in different intensities of PA (i.e., light, moderate, and vigorous), sedentary time, and step count measured using a tri-axial accelerometer and home BP on the same day, along with data comprising 115,575 records each of PA time, step counts, and home BP measurements. In the present study, we mainly found that longer time spent, especially in vigorous PA, and higher step count were associated with lower home BP levels. Analysis categorizing vigorous PA time with 0 min as the reference similarly showed associations of higher vigorous PA categories with lower BP levels. However, the observed effect sizes were small, indicating that while increased vigorous PA or step count may contribute to BP reduction, the overall impact is modest.

The results of the present study are in line with results from previous studies suggesting an association between higher questionnaire-assessed moderate-to-vigorous PA and lower office-measured BP [11, 12]. In addition, few studies have investigated the association of out-of-office BP with objectively measured PA [20]. To our knowledge, only a study in 660 participants from the electronic Framingham Heart Study with smartwatch-measured habitual PA and home BP measurements, reported that higher daily step counts were associated with lower home BP levels [20]. However, that study did not include time spent in different intensities of PA and did not assess PA and home BP on the same day. Our 5-year longitudinal study objectively measured PA using a tri-axial accelerometer and home BP on the same day, and suggests that longer time spent, especially in vigorous PA, and higher step counts are associated with lower home BP levels.

It is worth noting that sex and age differences were found in the association of PA, sedentary time, and step count and home BP. The associations of vigorous PA, sedentary time, and step count with home BP were more evident among men, potentially due to physiological differences, such as smaller hearts, lower blood volume, lower blood oxygen levels, and lower cardiovascular capacity in women [33, 34].

The association of moderate-to-vigorous PA, sedentary time, and step count with home BP was also more evident among participants aged <60 years, possibly due to age-related arterial stiffening. While PA is recommended for the prevention of hypertension across sex and all age groups [3–5], our results suggest that more PA time and less sedentary time for young-to-middle-aged adults, who have a higher lifetime risk for developing CVD and may benefit more from lifestyle modification.

Surprisingly, among women or participants aged ≥60 years in our study, light PA time was positively associated, while sedentary time was inversely associated, with home BP levels. In Japan, women or older age groups have a higher health literacy than men or younger age groups [35], which may encourage lifestyle changes like increasing light PA and reducing sedentary time, as an alternative to medical treatment, potentially resulting in reverse causality.

In the analysis stratified by antihypertensive medication use, light PA time was positively associated, while sedentary time was inversely associated, with home BP levels, especially among those not taking antihypertensive medication. Participants with relatively high BP levels (e.g., high normal, elevated, or grade 1 hypertension) who are not on antihypertensive medication may engage in lifestyle modification (e.g., less sedentary behavior or increased PA time) rather than initiating medical treatment, resulting in reverse causality. Current guidelines for the management of hypertension in Japan recommend lifestyle modification as the first line for managing high BP in participants with high normal or elevated BP, or grade 1 hypertension with few risk factors, before initiating antihypertensive treatment [3].

Obesity is a key risk factor for hypertension [3–5]. Light PA time was positively associated with BP levels, especially in participants with obesity. The effect of moderate or vigorous PA time on home BP tended to be larger in obese participants than in non-obese participants. The findings suggest the importance of a longer time in moderate-to-vigorous PA, especially for participants with obesity, to reduce body weight and lower home BP levels. The reasons for the differences in the association of PA with home BP between participants with different characteristics remain unclear and warrant further investigation.

In the stratified analysis by hypertension status, moderate or vigorous PA time showed a tendency for inverse associations with BP particularly among participants with hypertension. This may be because participants with hypertension benefit more from engaging in moderate or vigorous PA to lower home BP levels. On the other hand, light and moderate PA time tended to show positive associations with BP among participants without hypertension, possibly reflecting reverse causality, as these participants may engage in light-to-moderate PA as part of efforts to manage BP levels.

### Study limitations

The main strength of the present study is that the data on both monitor daily trends in home BP measured using Internet of Things (IoT) technologies and PA measured using a tri-axial accelerometer on the same day were collected during a 5-year study period, resulting in reliable analysis of the association of objectively measured time in different intensities of PA, sedentary time, step counts with self-measured BP at home. However, this study also has several limitations that should be considered. First, the participants recruited for the study were from a single local population of residents, which could impact the generalizability of the findings. Additionally, a relatively large number of participants were excluded (115/483, 23.8%) and differences in characteristics were observed between included and excluded participants. The excluded participants had higher BP and less PA or step counts, suggesting selection bias. Therefore, the participants included in the study may have been more health-conscious than those excluded. Second, the accelerometer used in this study cannot detect some types of PA (e.g., sedentary behavior and light PA) or posture accurately; therefore, time spent in sedentary behavior and light PA may be under or overestimated in cases when participants stand still for long hours [36]. Additionally, consistent with findings from previous studies reporting similarly minimal values [32, 37], although the median for vigorous PA was low, an inverse association between vigorous PA and home BP was identified in our study. Third, although statistical significance was found between step count and home BP, the clinical significance of the observed effect on home BP may be minimal. Finally, despite carefully controlled for major known confounders, the findings may be partly explained by differences in unknown confounders (e.g., dietary habits or socioeconomic factors).

## Conclusion

We found that longer time spent, especially in vigorous PA, and higher step counts were associated with lower home BP levels. The associations were more pronounced in men and participants aged <60 years. Furthermore, longer sedentary time was associated with higher home BP levels in men and participants aged <60 years. Given the small effect sizes observed in our study, the clinical significance of these associations remains unclear. However, our findings suggested a potential benefit of more PA and less sedentary time for the prevention and management of high BP, aligns with the new World Health Organization guidelines emphasizing the importance of attending to both PA and sedentary time to optimize the “balance” of these behaviors for better health [30].

## Summary

### What is known about the topic

- Most previous studies on physical activity (PA) and blood pressure (BP) have been based on self-reported questionnaires and office BP measurements.
- Few studies have examined the association between objectively measured PA and out-of-office BP.

### What this study adds

- Analyzed the association between self-measured home BP and objectively measured time spent in different PA intensities (light, moderate, vigorous), sedentary time, and step count, collected on the same day during a 5-year study period.
- Longer vigorous PA and higher step counts were associated with lower home BP, while sedentary time was associated with higher BP, particularly in men and participants aged <60 years.

## Supplementary information

Supplementary Table 1, Supplementary Table 2, Supplementary Table 3, Supplementary Table 4, Supplementary Table 5, Supplementary Table 6, Supplementary Table 7, Supplementary Table 8

## Data Availability

The datasets generated during and/or analyzed during the current study are available from the corresponding author on reasonable request.
