# Supplementary material for: The association between objectively measured physical activity and home blood pressure: a population-based real-world data analysis
Source: J Hum Hypertens. 2025 Apr 3;39(6):400–5. doi: 10.1038/s41371-025-01014-8 (PMC12151852; doi:10.1038/s41371-025-01014-8)
Supplement: Supplementary file 1 — Supplementary Table 1, Supplementary Table 2, Supplementary Table 3, Supplementary Table 4, Supplementary Table 5, Supplementary Table 6, Supplementary Table 7, Supplementary Table 8 [file 41371_2025_1014_MOESM1_ESM.doc]

**Supplementary Information**

**Supplementary Table 1.** Characteristics of participants in the Masuda Study, stratified by sex

**Supplementary Table 2.** Characteristics of participants in the Masuda Study, stratified by age

**Supplementary Table 3.** Association of vigorous PA time categories with home blood pressure

**Supplementary Table 4.** Association of physical activity, sedentary time, and step count with home blood pressure, stratified by sex

**Supplementary Table 5.** Association of physical activity, sedentary time, and step count with home blood pressure, stratified by age

**Supplementary Table 6**. Association of physical activity, sedentary time, and step count with home blood pressure, stratified by antihypertensive medication use

**Supplementary Table 7**. Association of physical activity, sedentary time, and step count with home blood pressure, stratified by obesity

**Supplementary Table 8**. Association of physical activity, sedentary time, and step count with home blood pressure, stratified by hypertension

**Supplementary Table 1.** Characteristics of participants in the Masuda Study, stratified by sex

| Variables | men  (n = 152) | women  (n = 216) | *P*-value |
| --- | --- | --- | --- |
| Age, years | 53.4 ± 11.7 | 54.1 ± 11.9 | 0.560 |
| Body mass index | 23.7 ± 3.1 | 22.4 ± 3.8 | <0.001 |
| SBP, mmHg | 121.2 ± 12.4 | 114.0 ± 14.5 | <0.001 |
| DBP, mmHg | 78.9 ± 8.9 | 73.3 ± 9.0 | <0.001 |
| Equipment time of tri-axial accelerometer, min/day | 728 (658−823) | 805 (714−899) | <0.001 |
| Light | 160 (120−207) | 200 (162−241) | <0.001 |
| Moderate | 76 (52−113) | 86 (63−115) | <0.001 |
| Vigorous | 1 (0−2) | 1 (1−2) | <0.001 |
| Sedentary time, min/day | 488 (417−560) | 509 (433−587) | <0.001 |
| Step count, day | 6445 (4523−9020) | 5828 (3982−8165) | <0.001 |
| Current smoker | 23 (15.1) | 6 (2.8) | <0.001 |
| Alcohol drinking | 115 (75.7) | 121 (56.0) | <0.001 |
| Antihypertensive medication use | 50 (32.9) | 46 (21.3) | 0.013 |
| Diabetes mellitus | 8 (5.3) | 12 (5.6) | 0.903 |

Data are presented as mean ± standard deviation, n (%), or median (interquartile range).

*SBP* systolic blood pressure, *DBP* diastolic blood pressure

**Supplementary Table 2.** Characteristics of participants in the Masuda Study, stratified by age

| Variables | age <60  (n = 240) | age ≥60  (n = 128) | *P*-value |
| --- | --- | --- | --- |
| Age, years | 47.0 ± 8.6 | 66.5 ± 4.1 | <0.001 |
| Women | 138 (57.5) | 78 (60.9) | 0.524 |
| Body mass index | 23.0 ± 3.7 | 23.0 ± 3.4 | 0.871 |
| SBP, mmHg | 115.0 ± 14.2 | 118.1 ± 14.0 | <0.001 |
| DBP, mmHg | 76.0 ± 9.9 | 74.3 ± 8.6 | <0.001 |
| Equipment time of tri-axial accelerometer, min/day | 777 (683−879) | 782 (695−875) | <0.001 |
| Light | 178 (136−225) | 197 (159−239) | <0.001 |
| Moderate | 81 (58−112) | 86 (60−118) | <0.001 |
| Vigorous | 1 (0−2) | 1 (0−2) | <0.001 |
| Sedentary time, min/day | 507 (433−586) | 494 (420−570) | <0.001 |
| Step count, day | 6224 (4414−8617) | 5767 (3897−8279) | <0.001 |
| Current smoker | 25 (10.4) | 4 (3.1) | 0.013 |
| Alcohol drinking | 162 (67.5) | 74 (57.8) | 0.065 |
| Antihypertensive medication use | 53 (22.1) | 43 (33.6) | 0.017 |
| Diabetes mellitus | 8 (3.3) | 12 (9.4) | 0.015 |

Data are presented as mean ± standard deviation, n (%), or median (interquartile range).

*SBP* systolic blood pressure, *DBP* diastolic blood pressure

**Supplementary Table 3.** Association of vigorous PA time categories with home blood pressure

|  | SBP (mmHg) | | DBP (mmHg) | |
| --- | --- | --- | --- | --- |
|  | *Β* (95% CI) | *P*-value | *Β* (95% CI) | *P*-value |
| Vigorous PA time, 0 min | Ref. |  | Ref. |  |
| 1 min | −0.06 (−0.20 to 0.07) | 0.351 | −0.11 (−0.19 to −0.03) | 0.011 |
| 2-4 min | −0.19 (−0.34 to −0.04) | 0.013 | −0.09 (−0.19 to 0.005) | 0.062 |
| 5 or more min | −0.30 (−0.51 to −0.08) | 0.007 | −0.20 (−0.33 to −0.06) | 0.005 |
| *P*-value for trend | 0.009 |  | 0.015 |  |

Adjusted by age, sex, body mass index, smoking status, alcohol drinking, diabetes mellitus, antihypertensive medication use, and sedentary time.

*CI* confidence interval*, SBP* systolic blood pressure, *DBP* diastolic blood pressure, *PA* physical activity

**Supplementary Table 4.** Association of physical activity, sedentary time, and step count with home blood pressure, stratified by sex

|  | Sex | SBP, mmHg | | P for interaction | DBP, mmHg | | P for interaction |
| --- | --- | --- | --- | --- | --- | --- | --- |
|  |  | Β (95% CI) | P-value |  | Β (95% CI) | P-value |  |
| Light PA time, hour | Men | −0.003 (−0.14 to 0.13) | 0.964 | <0.001 | −0.07 (−0.16 to 0.02) | 0.107 | <0.001 |
|  | Women | 0.29 (0.19 to 0.39) | <0.001 |  | 0.11 (0.05 to 0.17) | 0.001 |  |
| Moderate PA time, hour | Men | 0.15 (−0.02 to 0.32) | 0.078 | 0.049 | 0.02 (−0.09 to 0.13) | 0.728 | 0.001 |
|  | Women | 0.02 (−0.11 to 0.15) | 0.731 |  | −0.03 (−0.11 to 0.05) | 0.530 |  |
| Vigorous PA time, hour | Men | −1.90 (−2.59 to −1.21) | <0.001 | 0.001 | −1.04 (−1.50 to −0.58) | <0.001 | 0.023 |
|  | Women | −0.31 (−1.29 to 0.67) | 0.534 |  | −0.50 (−1.11 to 0.11) | 0.107 |  |
| Sedentary time, hour | Men | 0.16 (0.10 to 0.22) | <0.001 | <0.001 | 0.16 (0.12 to 0.20) | <0.001 | <0.001 |
|  | Women | −0.15 (−0.19 to −0.10) | <0.001 |  | −0.08 (−0.11 to −0.05) | <0.001 |  |
| Step count, 1000 steps | Men | −0.06 (−0.09 to −0.02) | 0.001 | <0.001 | −0.04 (−0.06 to −0.02) | 0.001 | <0.001 |
|  | Women | −0.03 (−0.05 to −0.003) | 0.030 |  | −0.01 (−0.02 to 0.01) | 0.483 |  |

We assessed 152 men and 216 women.

Analysis of moderate and vigorous PA time and step count was adjusted by age, body mass index, smoking status, alcohol drinking, diabetes mellitus, antihypertensive medication use, and sedentary time; analysis of light PA and sedentary time was adjusted by age, body mass index, smoking status, alcohol drinking, diabetes mellitus, antihypertensive medication use, and time spent in moderate and vigorous PA.

*CI* confidence interval, *SBP* systolic blood pressure, *DBP* diastolic blood pressure, *PA* physical activity

**Supplementary Table 5.** Association of physical activity, sedentary time, and step count with home blood pressure, stratified by age

|  | age group | SBP, mmHg | | P for interaction | DBP, mmHg | | P for interaction |
| --- | --- | --- | --- | --- | --- | --- | --- |
|  |  | Β (95% CI) | P-value |  | Β (95% CI) | P-value |  |
| Light PA time, hour | Age <60 | 0.29 (0.18 to 0.40) | <0.001 | 0.102 | 0.12 (0.04 to 0.20) | 0.003 | 0.734 |
|  | Age ≥60 | 0.18 (0.06 to 0.29) | 0.002 |  | 0.02 (−0.05 to 0.08) | 0.586 |  |
| Moderate PA time, hour | Age <60 | −0.18 (−0.32 to −0.04) | 0.012 | <0.001 | −0.14 (−0.24 to −0.04) | 0.007 | <0.001 |
|  | Age ≥60 | 0.14 (−0.01 to 0.29) | 0.059 |  | −0.01 (−0.09 to 0.08) | 0.851 |  |
| Vigorous PA time, hour | Age <60 | −3.55 (−4.38 to −2.71) | <0.001 | <0.001 | −1.91 (−2.50 to −1.33) | <0.001 | <0.001 |
|  | Age ≥60 | −0.40 (−1.16 to 0.35) | 0.293 |  | −0.53 (−0.97 to −0.10) | 0.016 |  |
| Sedentary time, hour | Age <60 | 0.11 (0.06 to 0.15) | <0.001 | <0.001 | 0.12 (0.08 to 0.15) | <0.001 | <0.001 |
|  | Age ≥60 | −0.16 (−0.21 to −0.10) | <0.001 |  | −0.08 (−0.11 to −0.05) | <0.001 |  |
| Step count, 1000 steps | Age <60 | −0.07 (−0.09 to −0.04) | <0.001 | 0.001 | −0.04 (−0.06 to −0.02) | <0.001 | <0.001 |
|  | Age ≥60 | −0.03 (−0.06 to 0.001) | 0.059 |  | −0.01 (−0.03 to 0.01) | 0.366 |  |

We assessed 240 participants aged <60 years and 128 participants aged ≥60 years.

Analysis of moderate and vigorous PA time and step count was adjusted by age, sex, body mass index, smoking status, alcohol drinking, diabetes mellitus, antihypertensive medication use, and sedentary time; analysis of light PA and sedentary time was adjusted by age, sex, body mass index, smoking status, alcohol drinking, diabetes mellitus, antihypertensive medication use, and time spent in moderate and vigorous PA.

*CI* confidence interval, *SBP* systolic blood pressure, *DBP* diastolic blood pressure, *PA* physical activity

**Supplementary Table 6.** Association of physical activity, sedentary time, and step count with home blood pressure, stratified by antihypertensive medication use

|  | antihypertensive medication use | SBP, mmHg | | *P* for interaction | DBP, mmHg | | *P* for interaction |
| --- | --- | --- | --- | --- | --- | --- | --- |
|  |  | *Β* (95% CI) | *P-*value |  | *Β* (95% CI) | *P-*value |  |
| Light PA time, hour | No | 0.21 (0.12 to 0.30) | <0.001 | 0.224 | 0.10 (0.04 to 0.16) | 0.001 | <0.001 |
|  | Yes | 0.17 (0.02 to 0.33) | 0.031 |  | −0.06 (−0.16 to 0.04) | 0.214 |  |
| Moderate PA time, hour | No | 0.09 (−0.03 to 0.20) | 0.141 | 0.032 | −0.02 (−0.09 to 0.05) | 0.591 | 0.011 |
|  | Yes | −0.11 (−0.33 to 0.10) | 0.302 |  | −0.09 (−0.22 to 0.04) | 0.193 |  |
| Vigorous PA time, hour | No | −1.63 (−2.23 to −1.03) | <0.001 | 0.543 | −0.99 (−1.38 to −0.60) | <0.001 | 0.348 |
|  | Yes | −2.02 (−3.28 to −0.76) | 0.002 |  | −1.26 (−2.04 to −0.47) | 0.002 |  |
| Sedentary time, hour | No | −0.04 (−0.08 to −0.002) | 0.039 | 0.012 | −0.03 (−0.05 to −0.002) | 0.034 | <0.001 |
|  | Yes | 0.01 (−0.05 to 0.08) | 0.697 |  | 0.11 (0.06 to 0.15) | <0.001 |  |
| Step count, 1000 steps | No | −0.05 (−0.08 to −0.03) | <0.001 | 0.570 | −0.02 (−0.04 to −0.01) | 0.001 | 0.510 |
|  | Yes | −0.04 (−0.08 to 0.002) | 0.059 |  | −0.02 (−0.05 to 0.001) | 0.059 |  |

We assessed 272 participants not taking antihypertensive medication and 96 participants taking antihypertensive medication.

Analysis for moderate and vigorous PA time and step counts was adjusted by age, sex, body mass index, smoking status, alcohol drinking, diabetes mellitus, and sedentary time.

Analysis for light PA and sedentary time was adjusted by age, sex, body mass index, smoking status, alcohol drinking, diabetes mellitus, and time spent in moderate and vigorous PA.

*CI* confidence interval, *SBP* systolic blood pressure, *DBP* diastolic blood pressure, *PA* physical activity

**Supplementary Table 7.** Association of physical activity, sedentary time, and step count with home blood pressure, stratified by obesity

|  | Obesity | SBP, mmHg | | *P* for interaction | DBP, mmHg | | *P* for interaction |
| --- | --- | --- | --- | --- | --- | --- | --- |
|  |  | *Β* (95% CI) | *P-*value |  | *Β* (95% CI) | *P-*value |  |
| Light PA time, hour | No | 0.12 (0.04 to 0.21) | 0.006 | 0.015 | 0.003 (−0.05 to 0.06) | 0.909 | 0.089 |
|  | Yes | 0.44 (0.26 to 0.62) | <0.001 |  | 0.18 (0.06 to 0.29) | 0.003 |  |
| Moderate PA time, hour | No | 0.07 (−0.05 to 0.18) | 0.250 | 0.283 | −0.01 (−0.08 to 0.06) | 0.773 | 0.030 |
|  | Yes | −0.16 (−0.41 to 0.09) | 0.220 |  | −0.20 (−0.37 to −0.04) | 0.015 |  |
| Vigorous PA time, hour | No | −1.46 (−2.04 to −0.88) | <0.001 | 0.178 | −0.92 (−1.29 to −0.55) | <0.001 | 0.059 |
|  | Yes | −2.27 (−4.13 to −0.40) | 0.017 |  | −1.55 (−2.76 to −0.34) | 0.012 |  |
| Sedentary time, hour | No | −0.03 (−0.07 to 0.01) | 0.163 | 0.688 | 0.01 (−0.02 to 0.03) | 0.585 | 0.197 |
|  | Yes | −0.08 (−0.16 to −0.01) | 0.031 |  | 0.01 (−0.04 to 0.06) | 0.634 |  |
| Step count, 1000 steps | No | −0.05 (−0.07 to −0.03) | <0.001 | 0.279 | −0.02 (−0.04 to −0.01) | 0.001 | 0.747 |
|  | Yes | −0.04 (−0.09 to 0.01) | 0.124 |  | −0.02 (−0.05 to 0.01) | 0.160 |  |

We assessed 269 participants who were not obese and 99 who were obese.

Analysis for moderate and vigorous PA time and step counts was adjusted by age, sex, body mass index, smoking status, alcohol drinking, diabetes mellitus, antihypertensive medication use, and sedentary time.

Analysis for light PA and sedentary time was adjusted by age, sex, body mass index, smoking status, alcohol drinking, diabetes mellitus, antihypertensive medication use, and time spent in moderate and vigorous PA.

*CI* confidence interval, *SBP* systolic blood pressure, *DBP* diastolic blood pressure, *PA* physical activity

**Supplementary Table 8**. Association of physical activity, sedentary time, and step count with home blood pressure, stratified by hypertension

|  | Hypertension | SBP, mmHg | | *P* for interaction | DBP, mmHg | | *P* for interaction |
| --- | --- | --- | --- | --- | --- | --- | --- |
|  |  | *Β* (95% CI) | *P-*value |  | *Β* (95% CI) | *P-*value |  |
| Light PA time, hour | No | 0.33 (0.16 to 0.50) | <0.001 | 0.277 | 0.27 (0.15 to 0.39) | <0.001 | <0.001 |
|  | Yes | 0.19 (0.10 to 0.27) | <0.001 |  | 0.02 (−0.04 to 0.08) | 0.499 |  |
| Moderate PA time, hour | No | 0.22 (0.01 to 0.43) | 0.043 | 0.567 | 0.19 (0.05 to 0.33) | 0.009 | 0.009 |
|  | Yes | −0.002 (−0.12 to 0.11) | 0.976 |  | −0.08 (−0.16 to −0.01) | 0.026 |  |
| Vigorous PA time, hour | No | −1.01 (−2.26 to 0.64) | 0.231 | 0.442 | −0.30 (−1.40 to 0.81) | 0.599 | 0.165 |
|  | Yes | −1.87 (−2.47 to −1.28) | <0.001 |  | −1.25 (−1.63 to −0.87) | <0.001 |  |
| Sedentary time, hour | No | 0.11 (0.03 to 0.18) | 0.005 | 0.095 | 0.07 (0.01 to 0.12) | 0.011 | 0.964 |
|  | Yes | −0.04 (−0.08 to −0.005) | 0.027 |  | 0.01 (−0.01 to 0.03) | 0.431 |  |
| Step count, 1000 steps | No | −0.03 (−0.07 to 0.01) | 0.194 | 0.670 | −0.01 (−0.04 to 0.02) | 0.487 | 0.299 |
|  | Yes | −0.05 (−0.08 to −0.03) | <0.001 |  | −0.03 (−0.04 to −0.01) | <0.001 |  |

We assessed 80 participants without hypertension based on home BP and 288 with hypertension.

Analysis for moderate and vigorous PA time and step counts was adjusted by age, sex, body mass index, smoking status, alcohol drinking, diabetes mellitus and sedentary time.

Analysis for light PA and sedentary time was adjusted by age, sex, body mass index, smoking status, alcohol drinking, diabetes mellitus and time spent in moderate and vigorous PA.

*CI* confidence interval, *SBP* systolic blood pressure, *DBP* diastolic blood pressure, *PA* physical activity
